# Development of PLA–Waste Paper Biocomposites with High Cellulose Content

**DOI:** 10.3390/polym16142000

**Published:** 2024-07-12

**Authors:** Concepción Delgado-Orti, Francisco J. Navas-Martos, Jose A. Rodríguez-Liébana, M. Dolores La Rubia, Sofía Jurado-Contreras

**Affiliations:** 1 Department of Chemical, Environmental and Materials Engineering, Campus Las Lagunillas, University of Jaén, 23071 Jaén, Spain; 2 Andaltec Technological Centre, Ampliación Polígono Industrial Cañada de la Fuente, C/Vilches 34, 23600 Martos, Spain; 3 University Institute for Research in Olive Grove and Olive Oil (INUO), Campus Las Lagunillas, University of Jaén, 23071 Jaén, Spain

**Keywords:** biobased, waste paper, biocomposite, cellulose, polylactic acid

## Abstract

In this study, the integration of paper industry waste with high cellulose content into biocomposites of polylactic acid (PLA), a widely used biobased polymer material, was investigated. The PLA/waste biocomposite samples (0–25 wt.%) were manufactured using the extrusion and injection moulding techniques. The mechanical test results showed improvements in terms of tensile properties and a decrease in impact strength as the percentage of residue increased. The melting temperature decreased, and the crystallinity increased in all biocomposites according to the Differential Scanning Calorimetry (DSC) analysis. Water absorption increased proportionally with the percentage of residue, attributed to the higher cellulose content in the biocomposites, determined by Fourier transform infrared spectroscopy (FT-IR) and X-ray diffraction (XRD) techniques. The scanning electron microscopy (SEM) fracture analysis demonstrated effective reinforcement–matrix cohesion, supporting the previously observed behaviour of the analysed materials. This work highlights the potential of using waste from the paper industry as reinforcement in PLA matrices, opening new perspectives for sustainable applications in the framework of the manufacture of composite materials.

## 1. Introduction

Currently, accelerated global change demands a balance between economic development and environmental preservation. While industries drive progress, the waste they generate poses economic and environmental challenges [1]. Effective waste management strategies, including cost reduction, recycling, and reuse, are essential to foster sustainability and promote a circular economy. These approaches minimise pollution and reduce reliance on landfills, thereby mitigating environmental impact and promoting resource efficiency [2].

The revalorisation of agricultural and industrial residues, such as fibres, husks, chips, and powder, presents innovative opportunities for recycling and reuse. Nowadays, there is a growing interest in using these residues as a potential source of reinforcements or filler in the manufacturing of polymer-based materials [3,4]. The addition of these fillers is often a simple and cost-effective strategy for producing bioplastics or biocomposites with improved properties [5]. When the source of waste to obtain the polymer-based material is derived from food scraps, biomass, municipal waste, or industry, such as residues from the paper industry, they are termed third-generation biopolymers. This generation is currently the most innovative and has great potential for future circular economy systems based on plastic materials [6].

Globally, between 7 and 10 billion tonnes of global waste are generated every year, 21% of which is industrial waste. Therefore, it is essential to find potential uses for these wastes while mitigating the environmental impact. Previous studies reported the feasibility of reusing industrial residues for the production of biocomposites, such as marble dust [7], fly ash [8], and red mud from the aluminium industry [9], among many others.

The average global consumption of paper per capita in 2020 was 52 kg/inhabitant, which means about 414.2 million tonnes of waste generated from the paper industry [10]. According to the Spanish National Statistics Institute (INE), in 2020, more than four million tons of paper and cardboard waste were generated only in Spain [11]. These types of waste contain a high percentage of lignin, cellulose, and hemicellulose as well as polymeric substances and microbial cells [5,12]. Most waste paper residues contain relatively intact fibres suitable for use in recycled paper or in the production of composite materials. Ye H. et al. (2023) employed paper waste and PVC powder to manufacture high-performance biocomposites [13].

Nowadays, there is great interest in the development of new, more environmentally friendly polymer composites reinforced with natural fibres or materials that originally come from waste [5,14]. Typically, the content of reinforcement can reach up to 50 wt.%. The criteria for the selection of the type and percentage of reinforcement include minimum values of tensile strength, toughness, stiffness, the elongation at break, adhesion between the fibre and polymer matrix, thermal stability, the dynamic and long-term behaviour of the composite, and processing costs [14,15].

The use of these types of reinforcements reduces the dependence on fossil sources to obtain polymeric materials, whose production has increased up to 460 million tonnes since the 1980s to 2021 [5,13]. However, their incorporation is a percentage, with the rest of the composite material often being fossil based. In this context, biopolymers are considered a suitable alternative as a polymer matrix due to their renewable origin and more environmentally friendly nature. Among the commercially available natural and synthetic biopolymers, one of the most interesting is poly(lactic acid) (PLA) [12,16]. Compared with other traditional bioplastics, PLA has excellent physical and chemical properties, meeting the requirements of applications such as interior and exterior decoration parts, automotive interior components, and architectural landscapes [17]. However, PLA also has some drawbacks that keep it from replacing conventional polymer matrices on an industrial scale, highlighting the high production costs [18]. Therefore, combining PLA with other biopolymers is one of the potential strategies to improve material properties and reduce manufacturing costs without affecting biodegradability. 

Cellulose, as the most abundant natural polymer, has been widely used as a component, filler, or processing additive in PLA-based composites [12,16,19]. The reinforcement of a polymer matrix with cellulosic fibres gives the resulting polymer composite good mechanical properties (stiffness, strength, and toughness) and generates low-cost and easy-to-dispose alternatives to other composite materials reinforced with synthetic glass fibres [15]. However, there is no extensive knowledge on the effect of incorporating waste from the paper industry, rich in cellulose, to reinforce PLA. 

In this work, PLA composite materials reinforced with waste from the paper industry, with high cellulose content, in different percentages were developed. The influence of this reinforcement on the mechanical, thermal, structural, and morphological properties of the resulting materials was evaluated. The development and characterisation of these innovative and environmentally friendly biocomposites provides crucial information for the creation of potential alternative routes of revalorising paper industry waste aligned with the global objectives of sustainability and the principles of circular economy [13].

## 2. Materials and Methods

### 2.1. Biocomposite Components

Ingeo™ Biopolymer 3251D PLA, supplied by NatureWorks (Minnetonka, MN, USA), was used as the polymeric matrix. This PLA has a value of 5.5 x104 g/mol in terms of average molecular weight (Mw), density of 1.24 g/cm^3^, MFI of 35 g/10 min (190 °C; 2.16 kg), a glass transition temperature of 55–60 °C, and low D-isomer content (99% l-lactide/1% D-lactide).

To improve the manufacturing process, a process additive (AD) (BYK P-MAX) based on a fatty acid ester was added. This additive was supplied by BYK-Chemie GmbH (Offenbach am Main, Germany) in the form of powder. 

The paper industry waste (WP) with a 74.6 wt.% of free cellulose content used was supplied by a paper and cardboard recycling industry located in Spain. The remaining composition of WP is biomass and plastic waste. This waste results from the rejection of the industrial production process after being subjected to a suitable washing and drying process. The WP samples collected were subjected to a milling process using a ZM 200 ultracentrifugal mill from RETSCH (Haan, Germany) with a 0.5 mm mesh sieve. The aim of this milling process is to reduce the WP particle size and increase its homogeneity. A 0.5 L solid sample was obtained after grinding for 5 min at a rotation speed of 18,000 rpm.

### 2.2. Biocomposite Manufacturing Process

Compounding procedure was performed on a Rehoscam internal mixer system (Scamex, Isques, France). The different components (PLA, WP, and AD) were mixed manually for 2 min prior to inserting it into the chamber of the internal mixer in batches of 150 g. A screw rotation speed of 60 rpm was used, establishing a material temperature of 173 °C and 7 min of mixing time. Before the mixing procedure, moisture was eliminated from the WP by storing it for 24 h in an air oven at 60 °C. Similarly, the PLA was also previously dried for 10 h at 60 °C, according to the supplier’s instructions, in a KKT 75 dryer from Werner Koch Maschinentechnik GmbH (Isprung, Germany).

After the compounding procedure, a solid mass was obtained, which had to be grinded to obtain the material in the proper format of flakes to be subsequently transformed into specimens using injection moulding technology. The grinding process was carried out utilising a WSGM-250 Purchades milling system (Madrid, Spain) with a 6 mm mesh size sieve.

**Figure 1:**
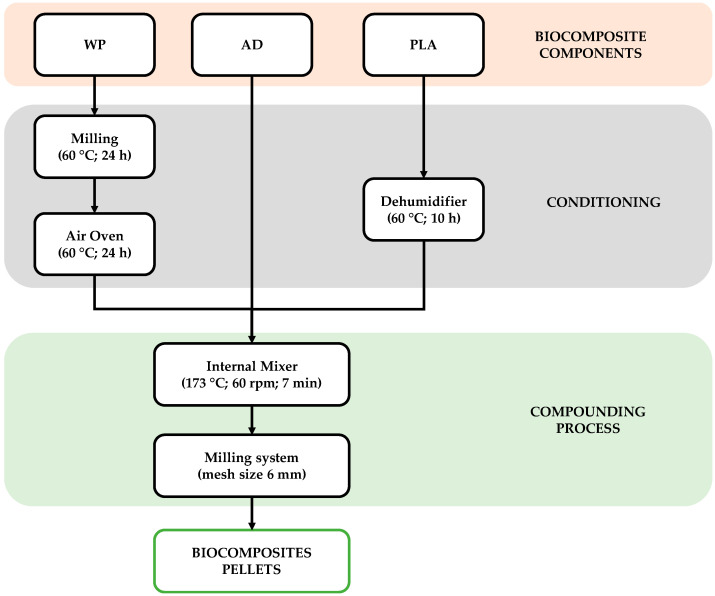
A scheme of the biocomposite manufacturing process.

The WP was incorporated into the PLA polymeric matrix in different percentages: 0, 5, 10, 15, and 25 wt.%. The AD was added at 1.5 wt.% in all cases. The denomination and content of the five biocomposites developed are presented in Table 1. Figure 1 shows a scheme of the biocomposite manufacturing process.

### 2.3. Characterisation of Components and Manufactured Biocomposites

#### 2.3.1. WP

The WP was analysed prior to incorporation into the biocomposite to determine the structure, thermal stability, and morphology. The structural and crystallographic composition were determined with X-ray diffraction (XRD) and Fourier transform infrared spectroscopy (FT-IR). A Tensor 27 FT-IR spectrometer (Bruker, Billerica, MA, USA) was employed for recording FT-IR spectra at 4 cm^−1^ resolution in the spectral range of 400 to 4000 cm^−1^ utilising the methodology of attenuated total reflectance (ATR). 

An Empyrean equipment containing a PIXcel-3D detector (PANalytical, Malvern, UK) was employed for the XDR analysis in the 2 theta range with 0.02 step size from 10 to 60°. The crystallinity index (CrI) was determined using the diffraction intensities of the amorphous and crystalline regions and by applying Equation (1):

(1) $$CrI\left(\%\right)=\frac{I_{200}-I_{am}}{I_{200}}\cdot 100$$

where *I*_200_ correspond to the top intensity of the specific diffraction peak associated with the grating (200) associated with the crystalline phase, which is located around 22°. The *I_am_* refers to the peak related to the amorphous phase and is located around 18° [20].

A high-resolution scanning electron microscope (FESEM) connected to Energy Dispersive X-ray Spectroscopy (EDS), model SM 840 from JEOL(Tokyo, Japan), was used to visualise the morphology.

Thermal stability was analysed by performing thermogravimetric tests using a TGA Q500 (TA Instruments, New Castle, DE, USA) under these specific conditions: a temperature from 10 to 1000 °C, a heating range of 10 °C/min, and a 50 mL·min^−1^ N_2_ flow rate. Derived thermogravimetry (DTG) curves were also made, obtaining the values of the degradation temperatures from them and the TG curves.

#### 2.3.2. Characterisation of Biocomposites

To assess the influence of adding of WP into the PLA matrix, the different biocomposites previously manufactured were characterised in terms of the mechanical, thermal, morphological, and structural properties. 

The mechanical properties were determined in terms of tensile and impact strength tests. Specimens recommended for the characterisation were manufactured with injection moulding technology according to the ISO 527-2 and 179-1 standards, respectively [21,22]. The equipment used was a Victory 28 injection moulding machine (Engel Holding GmbH, Schwertberg, Austria). Prior to the injection moulding, humidity was removed from the biocomposite pellets in the KKT 75 dryer (60 °C, 10 h). A 10 KS universal testing machine (Tinius Olsen, Redhill, UK) was used to determine tensile properties according to the ISO 527-2 standard. The impact resistance was determined in accordance with the ISO 179-1 standard using Charpy Impact Meter Izod IMPats 2281 (Metrotec, Lezo, Spain). Structural characterisation was carried out using FT-IR and XRD with the same equipment and conditions used for the WP and described in Section 2.3.1. The SEM coupled to the EDS equipment described in Section 2.3.1. was also used to analyse the fracture surface microstructure of the tested samples after determination of the impact strength.

The materials were also studied in terms of thermal behaviour in 822e Differential Scanning Calorimetry (DSC) equipment (Mettler Toledo, Greifensee, Switzerland) under the following conditions: heating mode, a heating ramp of 5 °C/min from 30 to 200 °C, followed by subsequent cooling up to 30 °C. From this analysis, the glass transition temperature (T_g_); the melting temperature (T_m_); the enthalpies corresponding to the melting and glass transition processes, ΔH_m_ and ΔH_g_, respectively; and the percentage of crystallinity (W_c_) of all the materials manufactured were obtained for all manufactured materials. Wc was calculated using Equation (2):

(2) $$W_{c}\left(\%\right)=\frac{{\Delta H}_{m}}{{\Delta H}_{mc}}\cdot \frac{1}{f_{PLA}}\cdot 100$$

The melting enthalpy, Δ*H_mc_* (J/g), corresponded to a sample of 100% crystalline PLA (93.1 J/g) [16] and *f_PLA_* the weight fraction of PLA in the biocomposite.

The water absorption capacity (*c*) was also evaluated using samples with dimensions 80 × 10 × 4 mm. The methodology used, immersion in distilled water, is detailed in in ISO 62 standard [23]. Equation (3) was used to determine the *c* values:

(3) $$c\left(\%\right)=\frac{m_{2}-m_{1}}{m_{2}}\cdot 100$$

where *m*_1_ is the original mass of the sample prior to the immersion (mg) and *m*_2_ is the final mass of the sample at the end of the immersion procedure.

## 3. Results

### 3.1. WP Characterisation

#### 3.1.1. Morphology

**Figure 2:**
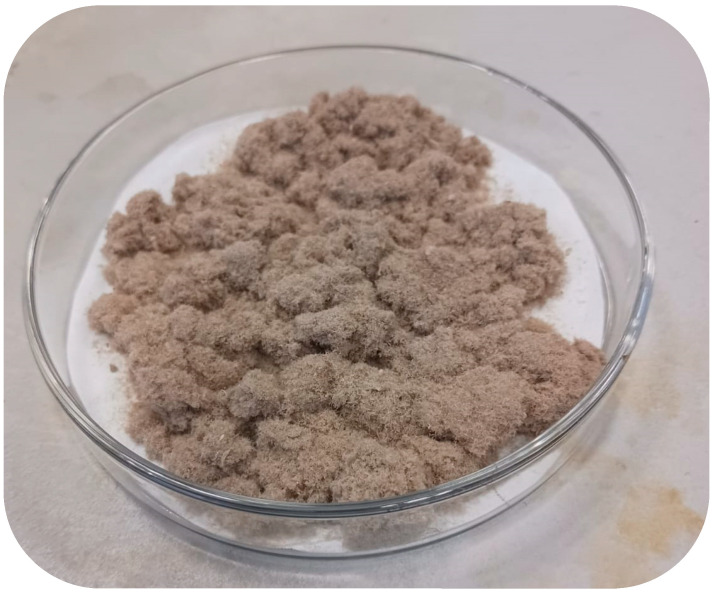
The appearance of a WP sample after the grinding process.

Figure 2 shows the WP used for the manufacturing of the different biocomposites.

**Figure 3:**
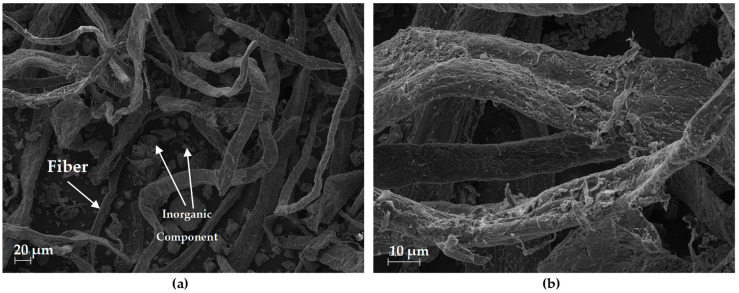
SEM images obtained from the WP after the milling process: (**a**) 250×; (**b**) 1000×.

The SEM images obtained from the WP are shown in Figure 3. As can be seen, the WP residue is composed of elongated fibres and inorganic compounds in granular form. The fibres present a uniform surface. Zhang et al. (2020) reported a similar structure that was identified for the paper residue from Chuanxing paper mill [24].

#### 3.1.2. FT-IR

**Figure 4:**
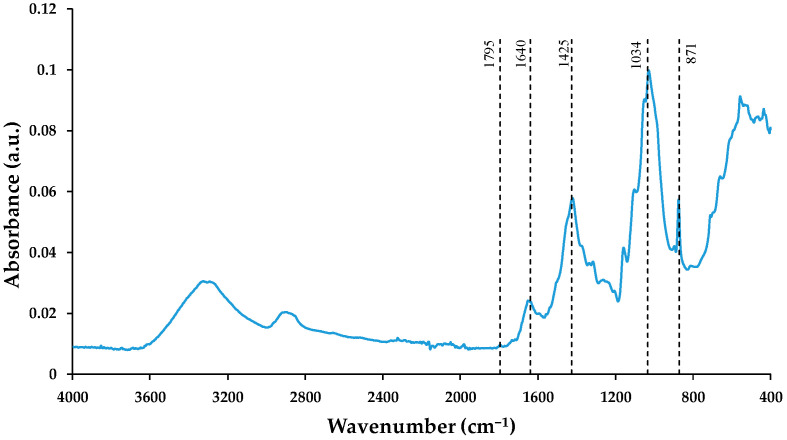
WP FT-IR spectra.

The results of the FT–IR analysis of the WP are shown in Figure 4. The region between 3300 and 3200 cm^−1^ was associated with the –OH groups of cellulose [4,17,25]. The peak at 1640 cm^−1^ associated with the O–H bending vibration of the absorbed water was evident in the spectra. This strong O–H bending vibration indicates a very strong WP polarity [24,26]. On the other hand, the presence of cellulose in the residue was identified through the absorption peak at 871 cm^−1^ corresponding to the β-glucose bond. The absorption peak at 1034 cm^−1^ was attributed to the stretching vibration of the C–O single bond and the strain vibration of the –OH present in cellulose [1,9]. The peak at 2918 cm^−1^ was due to stretching vibrations of saturated aliphatic C–H of the cellulose [1,17,26]. Therefore, it can be concluded that the main component of organic matter in WP is cellulose.

The peaks at 1425 and 1795 cm^−1^ indicate the presence of calcite (CaCO_3_) in the WP. This component is usually added in the paper industry between 20 and 30 wt.% [27]. The peak at 1425 cm^−1^ represents the C=O stretching vibration that is attributed to the internal vibrations of the CO_3_^2−^ structural unit, while the peak at 1795 cm^−1^ corresponds to the asymmetric C–O stretching vibration [28,29].

#### 3.1.3. XRD

**Figure 5:**
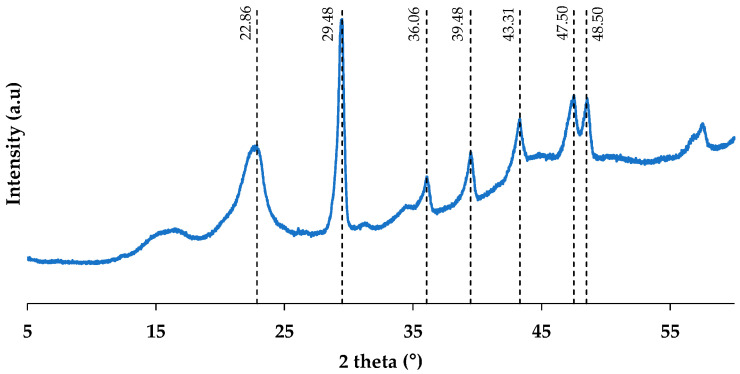
WP XRD pattern.

The XRD pattern obtained is shown in Figure 5. The diffraction pattern exhibits characteristic peaks of calcite (CaCO_3_) at 2θ angles 29.48, 36.06, 39.48, 43.31, 47.5, and 48.5°, corresponding to the crystallographic planes (104), (110), (113), (202), (018), and (116) [9,28]. The presence of this compound in paper residues has been corroborated by other authors. In the study by Xuan et al. (2020), the presence of calcite in paper residues was confirmed using XRD and XRF [9].

The diffraction planes around 15.2, 17.1, 22.8, and 34.8°, which corresponded to typical reflection planes of (110), (200), and (004), respectively, are associated with the different polymorphs of cellulose, characteristic of lignocellulosic residues [4,13,25,30,31,32]. Tingju Lu et al. (2014) observed the presence of cellulose at 22.5° in bamboo fibres, corresponding to the polymorphic structure of cellulose type I [33].

Additionally, the crystallinity index was calculated, which is highly important in natural reinforcements for polymeric matrices because it will confer strength and stiffness to the biocomposites [34]. A crystallinity of 59.62% was obtained for the WP residue. This value is comparable to the value obtained by Ye et al. (2023) for the paper residue, obtaining a value of 70.10% [13].

#### 3.1.4. Thermal Analysis

**Figure 6:**
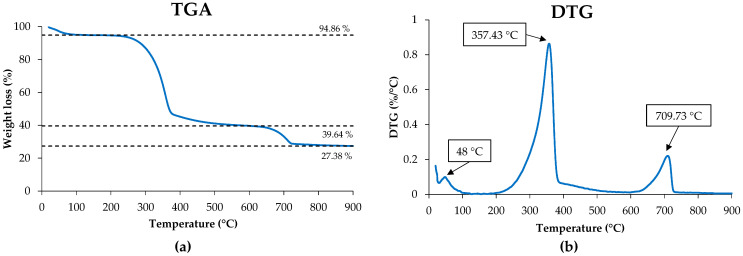
Thermal properties of WP: (**a**) TGA and (**b**) DTG curves.

The thermograms obtained from the TGA analysis are shown in Figure 6. The degradation of WP occurred in three phases. The first one, below 100 °C, was due to the loss in moisture and volatile components, resulting in a mass loss of up to 5.14 wt.% [18,35]. The second phase, located between 200 and 400 °C, is where the highest weight loss took place (up to 49.67 wt.%) as a consequence of the degradation of cellulose and hemicellulose [18,36]. The last phase, between 650 and 750 °C, presents a weight loss of 12.30%, which was attributed to CaCO_3_. The study by Karunadasa et al. (2019) reported that the thermal decomposition range of CaCO_3_ is located between 700 and 800 °C, so the last degradation peak can be attributed to this inorganic compound [37]. Finally, the high residual mass value (27.38 wt.%) was associated with the presence of inorganic components in WP. The DGT curve (Figure 6b) shows (a) the peak corresponding to water and volatile components (48 °C); (b) the degradation peaks assigned to cellulose at 357.48 °C, which were also reported by other authors [17]; and (c) the peak associated with the degradation of CaCO_3_ at 709.73 °C. 

### 3.2. Characterisation of Biocomposites

#### 3.2.1. FT-IR

**Figure 7:**
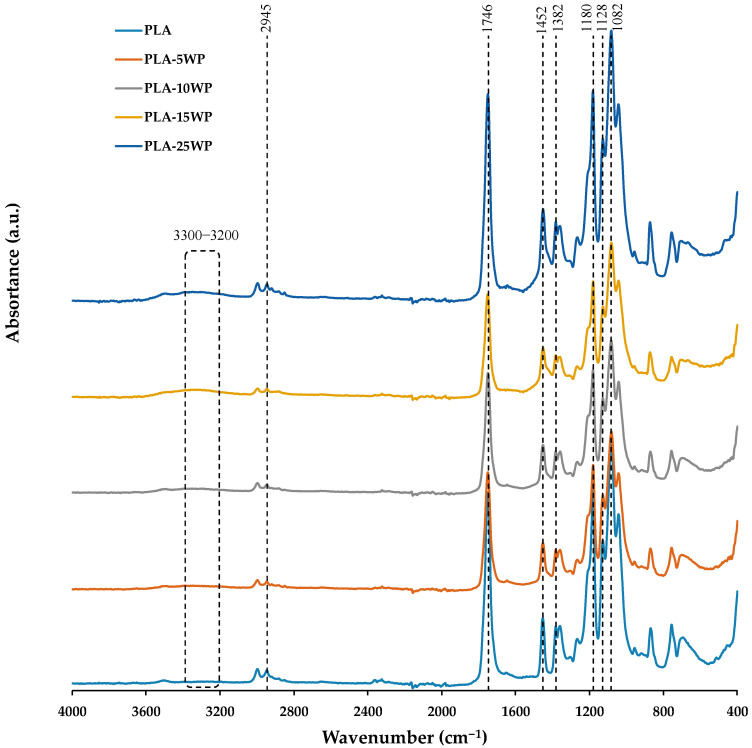
The FT-IR spectra of the PLA and biocomposites.

The chemical structure of PLA and biocomposites was explored using FT-IR studies. The resulting spectra are shown in Figure 7. For all materials, typical PLA absorption peaks were observed at 3657 and 3502 cm^−1^, corresponding to the –OH stretching vibration. The C–H stretching was recorded at 2994, 2944, and 2880 cm^−1^. The strong band at 1735 cm^−1^ attributed to the C=O vibration corresponds to the organic acids and ester present in PLA [17,25,33]. The characteristic absorption bands of pure PLA were observed at 1450, 1180, 1080, and 869 cm^−1^. The stretching vibration of the C–O groups was represented by the bands at 1180 and 1080 cm^−1^ [1].

Regardless of the percentage of WP, the position of the peaks remains unchanged. No clear increase was observed in the absorption peak at 1746 cm^−1^ after WP incorporation due to the symmetric C=O stretching present in cellulose and hemicellulose due to an overlap with PLA [12]. However, an increase in the intensity of the –OH band located between 3300 and 3200 cm^−1^ was registered with WP incorporation and could indicate that the WP reinforcement and the PLA matrix are interacting [33].

#### 3.2.2. XRD

**Figure 8:**
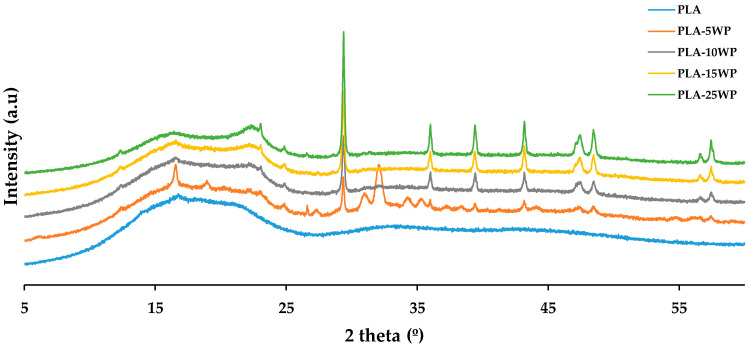
XRD patterns of PLA and biocomposites.

The crystalline characteristics of the WP-reinforced PLA biocomposites were examined in terms of XRD patterns (Figure 8). The biocomposites showed a typical structure of a PLA, with the XRD pattern modifying slightly after adding the WP, which indicates that the incorporation of the residue did not change the structure of the polymer matrix. The same effect was also reported by other authors when incorporating paper waste into a high density polyethylene (HDPE) matrix and incorporating virgin bamboo cellulosic fibres into PLA, observing that the structure of the polymer matrix used in each case remained unchanged [24,33]. An increase in intensity was observed in the peak associated with the WP, specifically at 22° due to the presence of cellulose [38]. The increase in this band when incorporating the WP can be attributed to an increase in the crystallinity of the biocomposite materials. The WP reinforcement acts as a nucleation agent, producing the formation of crystals, which overlap each other, resulting in a large number of small crystalline domains [25]. The peaks at 29.48, 36.06, 39.48, 43.31, 47.5, and 48.5° are identified with CaCO_3_ [28]. The intensity of all CaCO_3_ peaks increased as the percentage of WP incorporation increased.

#### 3.2.3. Mechanical Properties

**Figure 9:**
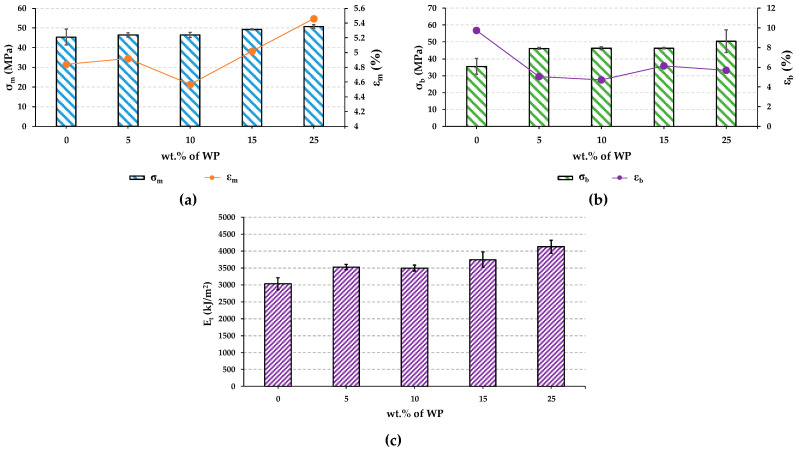
Tensile properties of PLA and biocomposites: (**a**) σ_m_ and ε_m_; (**b**) σ_b_ and ε_b_; (**c**) E_t_.

The tensile properties obtained for PLA and the manufactured biocomposites are shown in Figure 9: maximum tensile strength (*σ_m_*), tensile strength at break (*σ_b_*), tensile elongation (*ε_m_*), elongation at break (*ε_b_*), and Young’s modulus (*E_t_*). *σ_m_* increased with the percentage of WP (up to 11.8%) from 45.42 (PLA) to 50.77 MPa (PLA-25WP). A similar trend was observed for *σ_b_*, increasing up to 42.1% when incorporating 25 wt.% of WP, compared to PLA. This demonstrated that the incorporation of WP is beneficial in terms of tensile properties, which is the typical behaviour of those composites with uniformly and effectively distributed reinforcing particles, achieving a continuous phase for suitable stress transmission, and therefore proves a good dispersion of the reinforcement [24,25,39]. In addition, the large amount of inorganic mineral material contained in WP (Figure 3) can also explain the reinforcing effect of these particles in the polymer matrix [24]. On the other hand, the orientation of the fibres during the extrusion and injection moulding process can contribute to the increase in tensile properties [25].

In terms of *ε_m_*, a general increasing trend was observed from 4.84 to 5.46%. However, *ε_b_* decreased from 9.75% (PLA) to 4.7% (PLA-10WP) when adding 10 wt.% of WP. The increase in *ε_m_* may be due to the good alignment and dispersion of the fibres in the matrix [25,39]. Although ductility decreased, the increase in strength contributed to the higher toughness of the biocomposite since a balance between toughness and ductility is always reached in these types of materials [25]. La disminución de la ductilidad se ha reportado por Jałbrzykowski et al. (2020) al incorporar un 10 wt% de buckwheat husk fillers into teh PLA matrix [40].

As for *E_t_*, a value of 3033.25 MPa was recorded for PLA. When incorporating 5 wt.% of WP, it increases up to 3528.68 MPa (16.33%). This value was maintained for PLA-5WP and PLA-10WP and increased significantly with the addition of 15 and 25 wt.% of WP, reaching the maximum (4128.54 MPa) with the higher percentage of the WP, which means an increase of 36.11% regarding PLA. The increase in *E_t_* means that WP particles limited the mobility and deformability of the polymer chain, as corroborated by the decrease in elongation (Figure 9b) [15,24]. This behaviour in terms of stiffness is largely due to the fact that the cellulose contained in WP is composed of an amorphous part and a crystalline part, whose elastic modulus is around 130–240 GPa and thus induces stiffening to the final composite [39].

**Figure 10:**
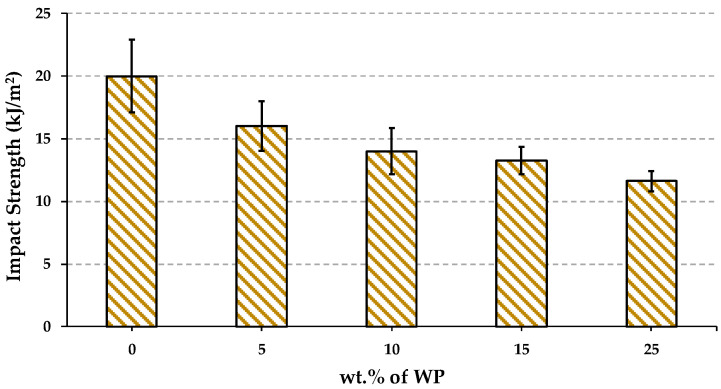
Impact strength of PLA and biocomposites.

Figure 10 shows a comparison of the values obtained from the Charpy impact strength tests carried out on the different materials. According to the results, the impact strength decreases with the incorporation of WP. Generally, the ability to absorb impact energy in composite resides in the polymer matrix, except in the case of brittle polymers such as PLA, whose fracture propagation energy is lower compared to the total energy required to initiate the fracture of the sample [18]. The presence of the WP generated stress concentrators at the reinforcement–matrix interface, leading to a reduction in impact strength [41], this effect being previously reported in biocomposites reinforced with natural fibres [42]. 

#### 3.2.4. SEM

**Figure 11:**
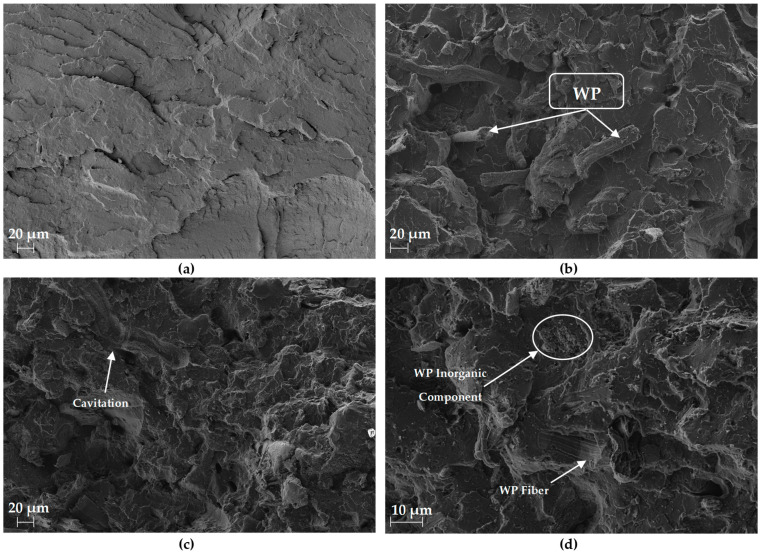
SEM images obtained from (**a**) PLA; (**b**) PLA-10WP; and (**c**) PLA-25WP. In addition, (**d**) PLA-25WP details.

The SEM images obtained from the fracture surface of the PLA and biocomposite specimens with 10 and 25 wt.% of WP subjected to the impact test are shown in Figure 11. All the samples analysed presented smooth fracture surfaces, and no WP agglomeration was detected, indicating suitable interfacial interaction between the WP and PLA matrix. This good dispersion of the WP corroborates the higher tensile strength of the biocomposites [39]. Tingju Lu et al. (2014) reported that better compatibility of bamboo cellulose fibres with a PLA matrix led to a significant increase in mechanical properties, which was corroborated by SEM [33].

The zoomed SEM image in Figure 11d confirms the presence of the cellulosic fibres and inorganic compounds (Figure 3) that are part of the composition of the WP present in the biocomposites. Figure 11c shows a cavitation phenomenon resulting from the extraction of the fibres, which indicates a good stress transfer between the matrix and the fibres supporting the improvement in the tensile properties [24].

#### 3.2.5. Water Absorption

**Figure 12:**
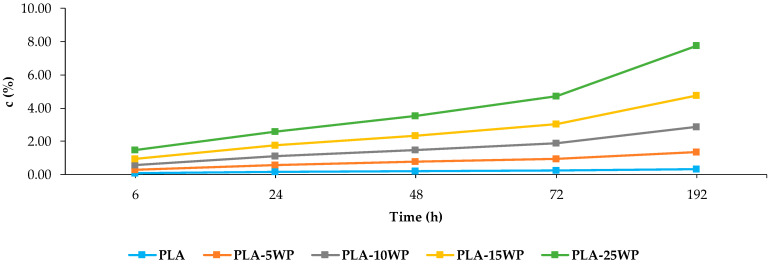
c of PLA and biocomposites.

As depicted in Figure 12, the amount of absorbed water increased continuously for PLA and the biocomposite samples. The molecular chain of cellulose fibre contains a large amount of hydroxyl groups (Figure 4), which are polar and give cellulose strong hydrophilicity. PLA is a high molecular weight linear aliphatic polyester polymer with a large amount of ester and methyl groups in its molecular chain, which is a non-polar molecule with strong hydrophobicity [17]. This is the main reason why PLA shows a lower water absorption capacity than the biocomposites with WP. Furthermore, the water absorption of the biocomposites increased with the higher WP content. The same trend was also observed by Ye et al. (2023), who reported that decreasing the PVC content in the biocomposites with WP increased the water absorption capacity of the materials [13]. This was attributed to the hydrophilic nature of the cellulose in the WP reinforcement, which features abundant hydroxyl groups that easily formed hydrogen bonds with water and therefore influence the polarity of the PLA chains, resulting in greater water absorption [25]. However, the increase in water absorption capacity was moderate, demonstrating a good interfacial bonding of the reinforcement and the polymer matrix because the PLA encloses the reinforcement, preventing water entry into the composite [17].

#### 3.2.6. DSC

**Figure 13:**
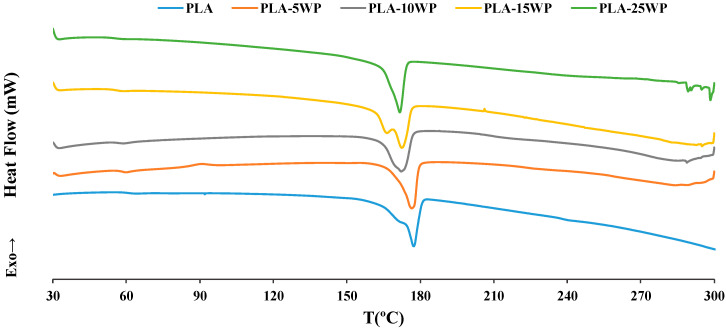
DSC curves of PLA and biocomposites.

Thermal properties of the PLA and biocomposites are shown in Figure 13 and Table 2. According to Figure 13, there is an endothermic region around 60 °C corresponding to the glass transition of PLA and a strong endothermic peak around 170 °C associated with the melting point of the polymer matrix [1,18]. A slight shift of the melting region to the left occurred when reinforcement was incorporated, this being more significant the higher the WP content. Garrido et al. (2023) reported a shift of the melting point peak towards lower temperatures when cork was incorporated into a PLA matrix [1]. On the other hand, a double melting peak was observed in the composites, which according to Mondal et al. (2021) can be attributed to the presence of α’ and α crystals, indicating PLA polymorphism [30].

Table 2 shows that *T_g_* decreases slightly from 62 to 58 °C with the increase in the WP percentage. The decrease in *T_g_* indicates that by incorporating WP, the PLA molecules degraded into smaller ones, these small molecules potentially acting as a plasticiser and therefore improving the toughness of the final biocomposites [33]. T_g_ results were comparable to those reported by Dewantoro et al. (2023) and Mondal et al. (2021) when incorporating cellulose nanocrystals (CNC) into a PLA matrix [25,30]. On the other hand, a slight increase in T_m_ and Δ*H_m_* was observed. The increase in *T_m_* indicates that the incorporation of WP increases the thermal stability of the final biocomposites [4]. This increase in *T_m_* is consistent with that observed in the study by Tao et al. (2021), an increase of 1.5 °C noticed when adding 15 wt.% of office paper waste in the PLA matrix [43]. Δ*H_m_* changed slightly, but the variation being less than 10 J/g indicated a maintenance of the crystalline structure of the material [18]. An increase in crystallinity from 17.8 to 23.6% was also reported, mainly due to WP acting as a nucleation agent for crystal growth [1,25]. Indeed, well-dispersed cellulose fibres are known to act as nucleating agents in the formation of new crystals in a variety of polymer matrices [5]. The presence of the WP was beneficial in the crystallisation process of the biocomposites, this same effect having been reported by Zhang et al. (2020) in a study in which it was observed that WP acted as a starting point for the formation of new crystals in the case of using two different polymeric matrices, one HDPE matrix and one LLPE matrix [24]. This increase in the crystallinity of the samples by incorporating WP also led to an increase in mechanical properties [33,40].

## 4. Conclusions

The characterisation of WP with SEM, FT-IR, and XRD confirmed the presence of a high cellulose content and showed a predominantly elongated fibrous morphology. The TGA analysis showed a high degradation temperature (357 °C), which made WP very promising as a reinforcement of polymeric matrices. The introduction of high percentages (up to 25 wt.%) of WP in a PLA matrix to obtain more sustainable biocomposites resulted in an increase in tensile properties. An increase of 11.8%, 42.1%, 12.8%, and 36.11% was reported for the maximum tensile strength, tensile strength at break, tensile elongation, and Young’s modulus, respectively. This means that WP showed good compatibility with the polymer matrix, resulting in good stress transfer and suitable behaviour as a potential reinforcement. SEM images confirmed good interaction between the reinforcement and the matrix and good dispersion, with no agglomeration observed. DSC curves showed slightly increased or maintained thermal stability and that WP acted as a nucleation agent, increasing the crystallinity of the final biocomposites up to 30.9%. Water absorption increased with higher percentage of WP incorporation due to the hydrophobicity of WP, presenting a higher number of –OH groups. 

According to the results presented above, it can be concluded that WP with high cellulose content has excellent characteristics to be considered as a potential reinforcement for PLA since its incorporation can result in (a) a decrease in material costs due to savings in PLA, (b) more sustainable and competitive biocomposite materials, and (c) the extension of the range of potential applications of PLA materials.

## Figures and Tables

**Table 1:** Content of PLA-based biocomposites (wt.%).

Reference	PLA	WP	AD
PLA	98.5	-	1.5
5WP/PLA	93.5	5	1.5
10WP/PLA	88.5	10	1.5
15WP/PLA	83.5	15	1.5
25WP/PLA	73.5	25	1.5

**Table 2:** Thermal properties obtained from PLA and biocomposites.

Reference	T_g_ (°C)	T_m_ (°C)	ΔH_m_ (J/g)	ΔH_g_ (J/g)	W_c_ (%)
PLA	62.51	170.60	36.18	1.66	17.83
5WP/PLA	59.75	176.74	44.15	0.72	21.98
10WP/PLA	59.26	172.45	45.43	0.98	23.09
15WP/PLA	58.64	172.96	45.41	0.69	23.58
25WP/PLA	58.30	172.23	44.96	0.25	23.34

## Data Availability

All data generated or analysed during this study are included in this published article.
